# Metal–Semiconductor Behavior along the Line of Stacking Order Change in Gated Multilayer Graphene

**DOI:** 10.3390/ma17081915

**Published:** 2024-04-21

**Authors:** Włodzimierz Jaskólski

**Affiliations:** Institute of Physics, Faculty of Physics, Astronomy and Informatics, Nicolaus Copernicus University in Toruń, Grudziądzka 5, 87-100 Toruń, Poland; wj@fizyka.umk.pl

**Keywords:** multilayer graphene, topological states, defects in graphene

## Abstract

We investigated gated multilayer graphene with stacking order changes along the armchair direction. We consider that some layers cracked to release shear strain at the stacking domain wall. The energy cones of graphene overlap along the corresponding direction in the *k*-space, so the topological gapless states from different valleys also overlap. However, these states strongly interact and split due to atomic-scale defects caused by the broken layers, yielding an effective energy gap. We find that for some gate voltages, the gap states cross and the metallic behavior along the stacking domain wall can be restored. In particular cases, a flat band appears at the Fermi energy. We show that for small variations in the gate voltage, the charge occupying this band oscillates between the outer layers.

## 1. Introduction

Stacking-order domain walls in multilayer graphene are most usually considered along the zigzag direction. They can be created when some layers are stretched, corrugated, or delaminated along the armchair direction [1,2,3,4]. Stacking order change causes the appearance of topological states. When the multilayer is gated and a gap opens, the one-dimensional topological gapless states appear at valleys *K* and *K*′ separated by $\frac{4}{3}\frac{\pi }{a}$ [1,5,6,7,8]. The gapless states are then valley-protected as long as there is no valley mixing caused, for example, by atomic-scale defects. However, it was shown that even under strong defects like bonds breaking in some layers at the domain wall, some gap states that connect the valence band (VB) and conduction band (CB) continua persist, and the systems preserve metallic character along the stacking domain wall [9,10].

The situation is different if we consider the stacking domain wall along the armchair direction. This is because, firstly, along the armchair direction, the valleys appear at the same *K* = 0 [11]. Therefore, the gapless states, which have opposite slopes *E(k)* at each valley, overlap and may no longer be valley-protected. Secondly, such stacking change causes strong shear strain at the interface between different stackings, at least for narrow domain walls.

**Figure 1:**
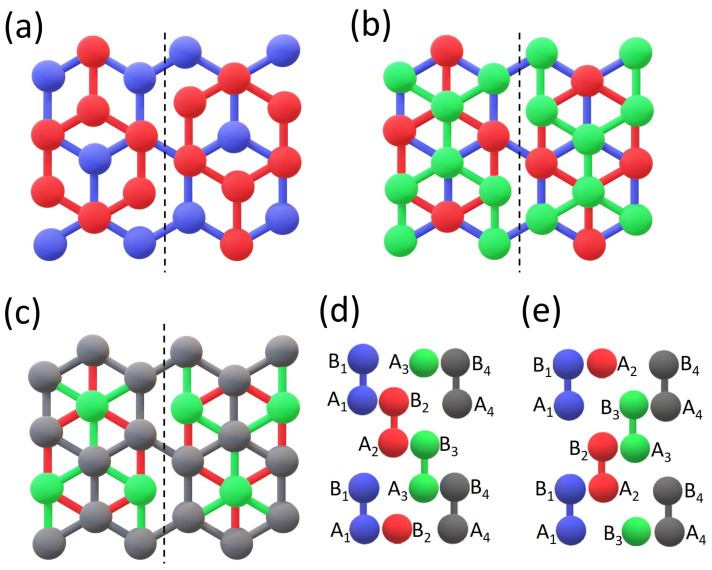
Schematic representations of the investigated systems. (**a**) BLG with stacking order change AB/BA. (**b**) TLG with stacking orders ABC/BAC. (**c**) FLG with stacking order change ABCA/BACB. Vertical broken lines mark the interface between different stackings. (**d**,**e**) Lateral views of sequences of carbon atoms in the consecutive layers along the armchair direction on both sides of the stacking domain wall. The layers from bottom to top are marked in colors: blue, red, green, and dark gray; they are also marked as indices of the A and B sublattices. In (**d**,**e**), the bottom layer is on the left. The bottom (blue) layer in (**c**) is covered and not seen.

In this work, we investigate the behavior of gapless states in such systems. We consider multilayers in which the interface bonds are broken in some layers to release any shear strain in the system. Figure 1 shows how unstrained bilayer (BLG), trilayer (TLG), and four-layer graphene (FLG) with stacking domain walls along the armchair direction can be formed. One can easily imagine that such cracks of layers may be present in realistic multilayers [12,13]. The bonds broken in some layers act as atomic-scale defects. Therefore, the gapless states strongly interact and split, yielding the appearance of the effective energy gap and thus the semiconducting properties of such multilayers. However, we show that for some gate voltages, some gap states cross and the energy gap disappears. The metallic behavior along the stacking domain wall can be restored. Transitions from semiconductor to metal can be achieved by changing the gate voltage. Moreover, for some gate voltages, the separated gap states in FLG connect at the Fermi level (*E_F_*), leading to a kind of flat band at *E_F_*, with charge occupying this band localized mainly in the outer layers.

## 2. System and Method

We investigated Bernal stacked bilayer and rhombohedral four-layer graphene with stacking-order domain walls. Figure 1 demonstrates how the consecutive layers are deposited. Panels (a) and (c) show the investigated BLG and FLG systems, and panel (b) shows an intermediate system of the TLG. Only small fragments of the multilayers are shown, while the investigated systems are infinite in both in-plane directions. The bottom layer is always uniform graphene, as well as the top layer in the case of FLG. The nodes of the FLG top layer have the same in-plane positions as the nodes of the bottom layer. The remaining layers are broken; they consist of two nonconnected half-infinite graphene layers shifted relative to each other by C-C along the armchair direction. This allows for the formation of a strainless interface with different stacking orders of multilayers on both sides. Note that due to the presence of the stacking domain wall, the systems studied are periodic only in the direction defined by the domain wall, i.e., in the armchair direction.

In the case of BLG, there are only Bernal stackings AB and BA on both sides of the interface. In the AB stacking, the atoms of sublattice B of the upper layer lie above the atoms of sublattice A of the lower layer, and reversely in the BA stacking. In the case of TLG, the stacking is rhombohedral (also called ABC) [14,15,16,17], i.e., it is Bernal stacking between the neighbor layers, but the sublattices of the top layer do not have the same in-plane positions as the corresponding sublattices of the bottom layer (this is to distinguish from the ABA sequence). We call BAC the arrangement of layers on the other side of the interface. To keep the rhombohedral stacking in the case of FLG, the sublattices of the top layer must have the same in-plane positions as the sublattices of the bottom layer. We call such stacking ABCA or BACB, depending on the side of the stacking domain wall interface. Figure 1d,e depict the stacking order of layers along the armchair direction on both sides of the domain wall, respectively. Note that these are only schematic views, not corresponding to any particular armchair line seen in Figure 1a–c.

To investigate the electronic structure of the defined multilayers, we calculate the local density of states (LDOS) at the interface. The calculations are performed using the Green function matching technique for a system composed of a conductor connected to two semi-infinite leads [18]. In our case, the conductor consists of the interface region, as defined in Figure 1a–c, connected on the left- and right-hand sides to two semi-infinite multilayers. The local density of states is calculated as $LDOS\left(E\right)=-\frac{1}{\pi }Im[TrG_{C}\left(E\right)]$, where $G_{C}\left(E\right)$ is the interface Green function determined by the Hamiltonians of the conductor and the leads. The Hamiltonians are calculated using a tight-binding approximation (TB). This approach has proved to adequately model the electronic structure of graphene systems close to the Fermi energy. The Green function matching approach based on the TB approximation is particularly convenient for nonperiodic, infinite systems, to avoid very large supercells. This is the case of the systems studied in this work, where the multilayer leads extend to infinity along the zigzag direction on both sides of the stacking domain wall interface. The values of intra-layer and inter-layer hopping parameters are t_i_ = 2.7 eV and t_e_ = 0.27 eV, respectively, following [19,20,21,22]. Since the system is periodic in the armchair direction, the Hamiltonian matrices of the central region and of the leads, as well as the local density of states, are *k*-dependent, i.e., LDOS(*E*,*k*), where *k* is the wave vector corresponding to this periodicity. To calculate LDOS from the above formula, a small imaginary part is added to the energy, i.e., *E* + *iε*. The numerical calculations are performed with the use of the Netlib LAPACK library.

## 3. Results and Discussion

We perform calculations for defined graphene multilayers in a perpendicular external electric field. Several values of the field are considered, namely **E** = 7, 22, 44, and 120 mV/Å. Assuming the inter-layer distance, d = 3.35 Å, the values of **E** translate into gate voltages between the neighbor layers: *V* = 0.025, 0.075, 0.15, and 0.4 eV. The voltages we consider are available in the experiment and are of the same order as typically used for multilayer graphene systems [22,23,24].

As for the external electric field, it can be applied in different configurations of the gate electrodes [25] to study charge polarization in multilayers [26,27]. In graphene systems with stacking domain walls, the role of the external field is to open an energy gap and reveal the existence of topological gap states. In a tight-binding model, the field enters via *V_L_* added to diagonal elements of the Hamiltonian matrix of a given layer, *L*. We use such a gate configuration, in which $V_{L}=V\times (L-1)$, where *L* = 1 is the bottom layer. As for the gate voltage modulation, the reader is encouraged to consult [28].

**Figure 2:**
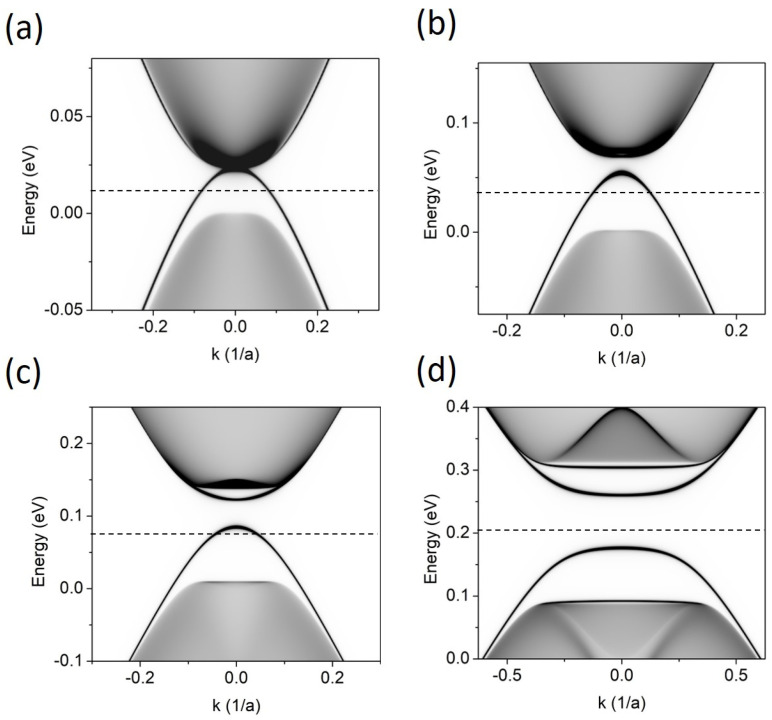
LDOS for BLG with stacking-order domain wall along the armchair direction, as visualized in Figure 1a, for different gate voltages (*V*) applied between the layers. (**a**) *V* = 0.025 eV, (**b**) *V* = 0.075 eV, (**c**) *V* = 0.15 eV, and (**d**) *V* = 0.4 eV. The Fermi level, marked as a dashed line, lies in the middle of the gap defined by the energy continua.

In Figure 2, we show the LDOS calculated in the BLG system for four values of the gate voltage, *V*. Of the four gapless states that may appear at all at *K* = 0 in the BLG with a stacking domain wall [11], there is little left in the energy gap. For *V* = 0.4 eV, four separated flat bands (two of them are situated very close to the band continua) are visible as a remnant of interacting topological gapless states. This is caused by the atomic-scale crack in the upper layer. For smaller *V,* fewer bands are visible in the energy gap. Finally, for *V* = 0.025 eV, the band that “grows up” from the VB continuum connects with the conduction band and the system takes on a metallic character along the domain wall.

**Figure 3:**
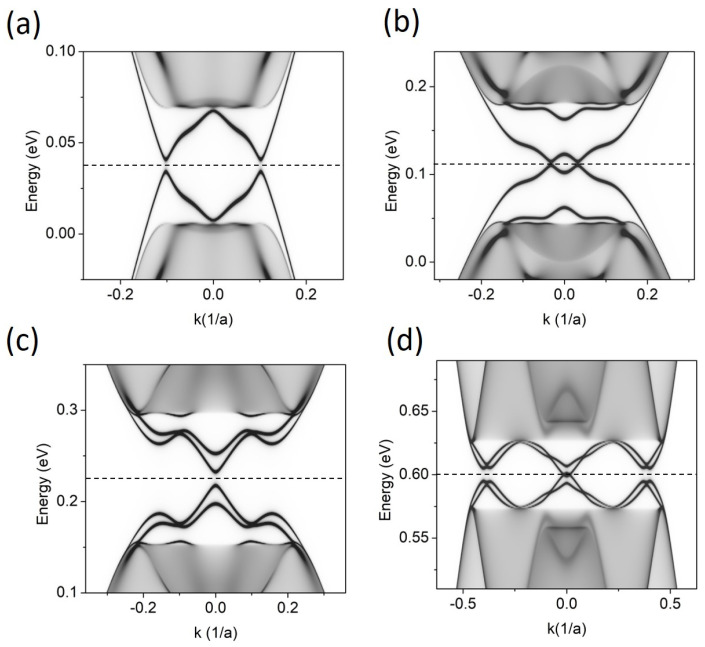
LDOS for FLG with stacking-order domain wall along the armchair direction, as visualized in Figure 1c, for different gate voltages, *V.* (**a**) *V* = 0.025 eV, (**b**) *V* = 0.075 eV, (**c**) *V* = 0.15 eV, and (**d**) *V* = 0.4 eV. The Fermi level, marked as a dashed line, lies in the middle of the energy gap.

Figure 3 shows the LDOS for an FLG system. Now, the situation is more complex. The stacking order change may, in principle, lead to as many as eight gapless states at *K* = 0, but here, the number of bands in the energy gap depends strongly on *V.* For very small *V* = 0.025 eV, the overall picture resembles two pairs of intersecting topological states with opposite slopes. They slightly repel at the crossing points, so a small gap opens at *E_F_*. For higher values, e.g., *V* = 0.075 eV, this small gap closes, the band crossing points approach the cone center, and two new bands detach from the VB and CB continua. When the gate increases further, the gap opens (for *V* ≈ 0.1 eV), but closes again for *V* higher than 0.3 eV, and the system restores its metallic character.

**Figure 4:**
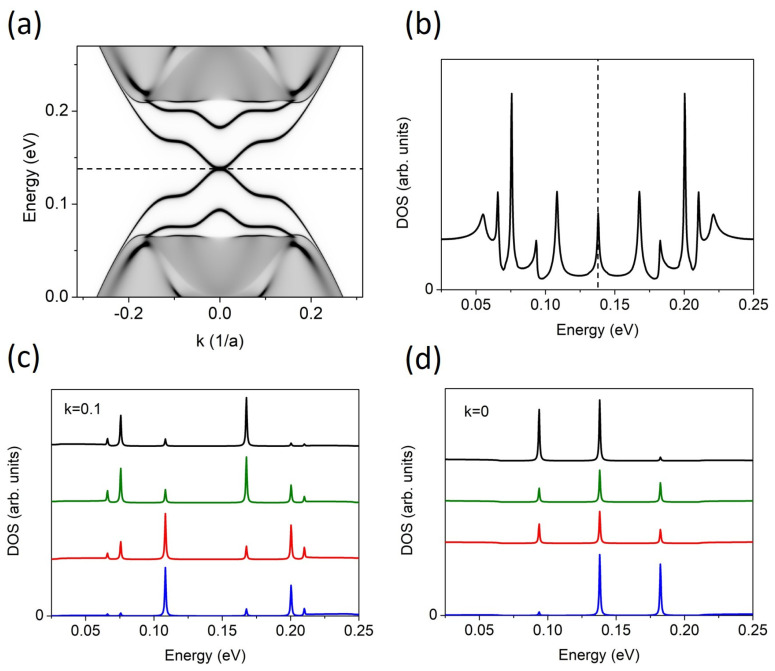
(**a**) LDOS for FLG and *V* = 0.092 eV. (**b**) PDOS, i.e., LDOS integrated over *k* in the IBZ. The Fermi level is marked as a dashed line. (**c**,**d**) LDOS distribution in the layers for *k* = 0.1 and *k* = 0, respectively. The density in consecutive layers, from bottom to top, is shown in colors: blue, red, green, and black.

Especially interesting is the case when *V* ≈ 0.092 eV. The corresponding LDOS is presented in Figure 4a. The four bands in the energy gap show flat areas, and a short flat (in the range of *k*) band is also formed at the Fermi level, where two bands touch. The increased density of this flat band at *E* = 0.14 eV is seen in Figure 4, where the projected density of states, i.e., $PDOS\left(E\right)=\int_{IBZ}^{\;}LDOS\left(E,k\right)dk$, is shown. The distribution of band densities in the layers is visualized in Figure 4c,d. Of the two bands that touch at the Fermi level, the band below *E_F_* is localized in the two lower layers, while the band above *E_F_* localizes in the two upper layers. Slightly away from the cone center, i.e., for *k* = 0.1, the distribution over layers is uniform. However, for *k* = 0, i.e., just at the flat band, a fraction of the charge moves to the outer layers. A similar effect of leakage of charge to the outer layers was also recently observed in [15] for a system of double twisted trilayers.

**Figure 5:**
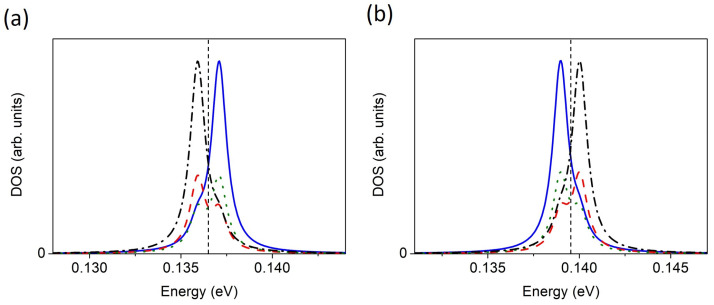
The LDOS(*E*,*k* = 0) distribution in the layers for FLG and two different gate voltages, *V.* (**a**) *V* = 0.091 eV; (**b**) *V* = 0.093 eV. The density in consecutive layers is shown as blue (solid), red (dashed), green (dotted), and black (dot-dashed). The Fermi level is marked as a vertical short-dashed line. The highest peak below the Fermi level corresponds to (**a**) the top layer and (**b**) the bottom layer.

At low temperatures, the flat band at *E_F_* is only half-filled. Note that in graphene systems, such a band can give rise to magnetic effects [17,29,30,31,32] and superconductivity [32,33,34,35,36]. The width of the flat band at *E_F_* is about 5 meV. In Figure 5, we show that the LDOS(*E*,*k* = 0) of this band resolved into the layers, for two values of the gate voltage, *V* = 0.091 eV and *V* = 0.093 eV, for which the flat band is still present at *E_F_*.This result confirms that at *k* = 0, the density is localized mainly in the outer layers (blue and black). More importantly, we find that a slight increase in V by only a few mV moves the charge occupying the flat band at *E_F_* from the top to the bottom layer.

**Figure 6:**
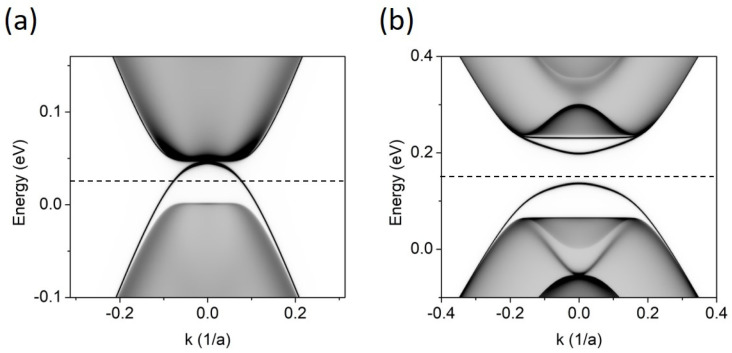
LDOS for TLG with stacking-order domain wall along the armchair direction, as visualized in Figure 1b, for different gate voltages (*V*) applied between the layers. (**a**) *V* = 0.025 eV, (**b**) *V* = 0.15 eV. The Fermi level is marked as a dashed line.

The calculations have also been performed for the trilayer (Figure 1b). Since the middle and top layers of the TLG are broken, they do not form a bilayer with a stacking domain wall. Therefore, the results do not differ substantially from those for BLG, and they are not discussed in detail. For illustration, they are presented in Figure 6 for two values of the gate voltage, *V* = 0.025 eV and *V* = 0.15 eV. We have also checked that wider separation of the half-graphene layers does not essentially change the results. The band crossing points and the separation of bands can occur for different voltages, but the overall picture of the gap states and their behavior with the changing *V* is similar.

## 4. Conclusions

We have studied the electronic structure of gated bilayer and four-layer graphene with stacking-order domain walls created along the armchair direction. Bernal and rhombohedral stackings have been considered for BLG and FLG, respectively. Since the change in stacking across the armchair direction causes shear strain at the domain wall, we allow some layers to crack, i.e., to break C-C bonds along the domain wall to release the strain.

We have investigated the local density of states along the domain wall, i.e., at the interface of the adjacent stacking orders. The graphene energy cones *E*(*k*) overlap in the corresponding direction in the *k*-space. Thus, all the possible topological gapless states appear at the same valley, *K* = 0. Since the broken bonds act as atomic-scale defects and lead to the removal of valley protection of gapless topological states, these states interact and split, yielding an effective energy gap. We have, however, found that for some gate voltages, the metallic character of the multilayers along the domain wall is restored. In the case of BLG, it happens for very small gates. In the case of the four-layer graphene, the gap states cross or anticross, i.e., they close or open the energy gap, depending on the gate.

In the case of four-layer graphene and for a specific value of the gate voltage, some bands in the gap connect, forming a kind of a short flat band at the Fermi energy. We have demonstrated that the density of this band is moved to the outer layers. Finally, we have also shown that for very small changes in the gate voltage, the charge that occupies the flat band at *E_F_* can change its localization between the bottom and the top layers. This gives us a tool for changing the layer localization of one-dimensional currents that, at *E_F_*, can flow along the stacking domain wall.

## Data Availability

Data are contained within the article. No new data were created.
